# Do Children Cause the Cognitive Stimulation they Receive? Modelling the Direction of Causality

**DOI:** 10.1007/s10519-024-10195-w

**Published:** 2024-09-10

**Authors:** Alexandra Starr, Olakunle Oginni, Sophie von Stumm

**Affiliations:** 1 University of York, York, UK; 2 King’s College London, London, UK; 3 Wolfson Centre for Young People’s Mental Health, Cardiff University, Cardiff, UK

**Keywords:** Cognitive development, Cognitive stimulation, Cross-lagged, Genetics, Polygenic scores

## Abstract

We tested the directionality of associations between children’s early-life cognitive development and the cognitive stimulation that they received from their parents. Our sample included up to 15,314 children from the Twins Early Development Study (TEDS), who were born between 1994 and 1996 in England and Wales and assessed at ages 3 and 4 years on cognitive development and cognitive stimulation, including singing rhymes, reading books, and playing games. Using genetically informative cross-lagged models, we found consistent, bidirectional effects from cognitive development at age 3 to cognitive stimulation at age 4, and from cognitive stimulation at age 3 to cognitive development at age 4. These cross-lagged longitudinal effects were largely explained by underlying common genetic and shared environmental factors, rather than reflecting causal mechanisms. Our findings emphasize the active role that children play in constructing their own learning experiences.

**Supplementary Information:**

The online version contains supplementary material available at 10.1007/s10519-024-10195-w.

## Introduction

**Fig. 1:**
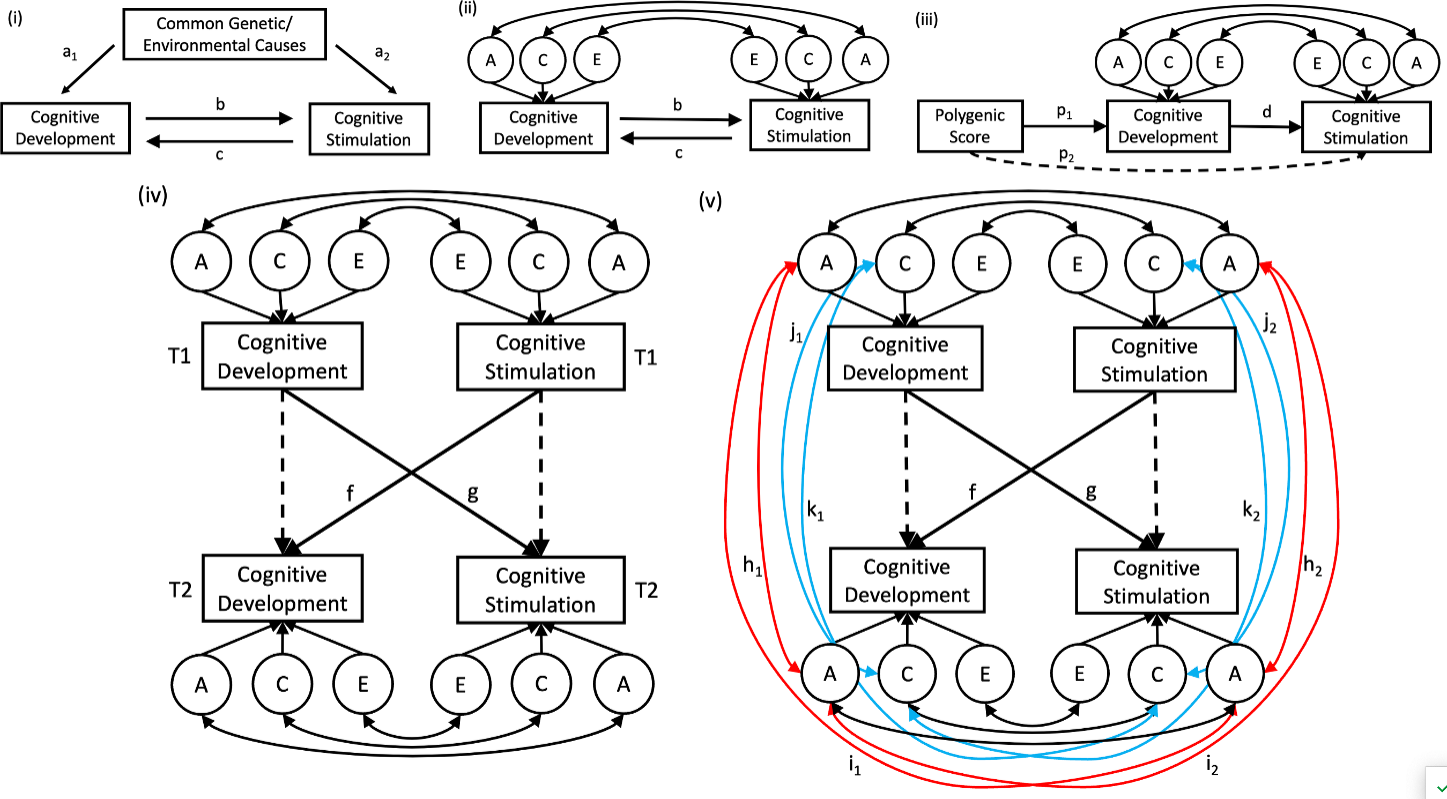
Genetically informed models for testing the direction of causality between cognitive development and cognitive stimulation *Note* Panel (**i**) illustrates that common causes can account for the relationship between cognitive development and cognitive stimulation (paths a1/2), or both constructs may be independently causally associated in either direction (b or c). Panel (**ii**) maps the Direction-of-Causality (DoC) model, which uses the cross-trait, cross-twin correlations of additive genetic (A), shared environmental (C), and non-shared environmental (E) factors to infer the direction of causality (b or c). The Mendelian Randomization (MR) of the DoC (panel (**iii**)) uses polygenic scores (PGS) as the instrument variable (p1) to test a unidirectional causal path, here from cognitive development to cognitive stimulation (d), while controlling for direct pleiotropy (p2, dotted arrow). The cross-lagged twin model (panel (**iv**)) tests causal influences across T1 and T2 (cross-lagged paths f and g) while controlling for the constructs’ stability (dotted arrows) and ACE correlations within timepoints. The extended cross-lagged twin model in panel (**v**) tests for the possibility that the direct, cross-lagged paths in panel (**iv**) can be attributed to cross-time correlations of genetic factors (rA in red colour; h1/2 and i1/2) and shared environmental factors (rC in blue colour; j1/2 and k1/2)

Children’s early-life cognitive development – the ability to think, reason, and learn – is a powerful predictor of later developmental outcomes like school performance and well-being (Feinstein and Bynner 2004; von Stumm et al. 2020). While the causes for children’s differences in cognitive development are manifold, a great focus has been on parents’ efforts to engage children in activities that promote learning, known as cognitive stimulation. Positive associations have been widely reported between children’s cognitive development and their engagement in cognitively stimulating activities, such as shared book reading (Xie et al. 2018), conversing with children (Hart and Risley 1995), and singing nursery rhymes together (Mullen 2017). However, the evolution of the association between cognitive development and cognitive stimulation remains unclear in two ways. First, we do not know to what extent cognitive development and stimulation may cause each other, rather than having a common origin, for example when the same genetic or environmental factors affect both constructs (cf. genetic confounding; Wertz et al. 2019; Fig. 1, panel (i). Many studies have shown that so-called environmental measures are genetically influenced and not truly exogenous to the individual (Dick 2011; Krapohl et al. 2017; Plomin 1995). Furthermore, the environmental and genetic factors that influence one phenotype tend to also influence related phenotypes (Avinun 2020). It follows that individual differences in phenotypically related constructs – like cognitive development and cognitive stimulation – often share common etiologies.

Second, if there is an independent causal relationship between cognitive development and stimulation, what is its direction? At a first glance, we might assume that cognitive stimulation should foster cognitive development, because cognitive stimulation offers opportunities for children to grow cognitively (Tucker-Drob and Harden 2012; Xiong et al. 2020). However, the reverse is also possible: children with better cognitive abilities may elicit or ‘cause’ greater cognitive stimulation from their environments. For example, precocious children might evoke more learning opportunities from their parents, like being taken to the library, visiting museums, or having frequent and challenging conversations.

Genetically informative study designs can help disentangle relationships between cognitive development and cognitive stimulation (McAdams et al. 2021). The classical twin design compares the phenotypic resemblance of monozygotic (MZ) twin pairs (who share 100% of their genome and are genetically identical) to that of dizygotic (DZ) twin pairs (who, on average, share 50% of their segregating genes) to estimate the respective contributions of genetic and environmental influences to phenotypic trait differences. Twin models can be used to decompose variance in phenotypic traits into additive genetic (A), shared environmental (C), and unique environmental influences (E; ACE models). An extension of the ACE model is the Direction of Causality (DoC) model that tests for bidirectional causal relationships between two constructs, if certain assumptions are met (Heath et al. 1993; Fig. 1 panel (ii). Specifically, DoC models can ascertain the direction of causality in cross-sectional data for two traits, whose mode of inheritance is sufficiently different (e.g., A on one trait is significantly greater or lower than on the other). For example, DoC models recently showed that 7-year-olds’ ability to read determines how much they choose to read, rather than the degree of print exposure determining their ability to read (van Bergen et al. 2018).

An extension of the DoC model is the Mendelian-Randomization Direction of Causation (MRDoC) model that includes polygenic scores (PGS) as an instrument variable in twin samples for whom genotype data are available (Minică et al. 2018; Fig. 1 panel (iii). This model tests unidirectional rather than bidirectional causality, while relaxing the assumptions of the DoC models, meaning that the mode of inheritance does not have to differ for both constructs. PGS aggregate thousands of DNA variants, whose associations with a target phenotype were identified in genome-wide association (GWA) studies, and index individuals’ genetic propensities for phenotypic development (e.g., Plomin & von Stumm, 2018). PGS can serve as instrument variables in MR models if they are plausible and significant predictors of the exposure, whose causal effect on an outcome is being tested (Sanderson et al. 2022; Davey Smith and Ebrahim 2003). Because no GWA study on children’s early life cognitive development has been published to date, studies that seek to capture children’s genetic propensities for cognitive development utilise PGS based on a GWA study for years spent in education (e.g., von Stumm et al. 2023). Educational attainment and cognitive traits are genetically correlated phenotypes (e.g., Calvin et al. 2012), even though the extent to which the DNA variants associated with years spent in education are identical with those for early life cognitive development is unclear. Thus, PGS for years spent in education can be used in an MRDoC model to test if cognitive development causes cognitive stimulation (Fig. 1, panel (iii). Because no GWA study has been reported for cognitive stimulation, testing the reverse direction of causality from cognitive stimulation to cognitive development is not possible (i.e., there is no PGS for cognitive stimulation).

A third option is the cross-lagged twin model (Burt et al. 2005), which tests the effect of a trait at an earlier assessment time (T1) on another trait at a later assessment time (T2; i.e., cross-lagged paths), while controlling for the constructs’ stability over time and their ACE factors’ correlations within the assessment times (Fig. 1, panel (iv). Cross-lagged twin models do not require the constructs to have different modes of inheritance, and they do not include instrument variables. Yet, they necessitate longitudinal data, with the two constructs of interest being assessed two or more times. The cross-lagged twin model can be further extended to control for the cross-time correlations of the genetic and shared environmental influences on cognitive development and cognitive stimulation. This extended cross-lagged twin model allows testing if the direct effects across time – that is, the longitudinal stability of each construct and cross-lagged effects between the constructs – are confounded by genetic and shared environmental factors that affect both constructs. In other words, this model (Fig. 1, panel (v) tests if genetic or shared environmental factors act as a common origin of cognitive development and cognitive stimulation regarding their cross-sectional as well as longitudinal relationship. Accounting for these potential confounding effects strengthens the rationale for inferring causal effects from the direct paths in the model compared to alternative approaches (Burt et al. 2005).

The aim of the current research was to clarify the direction of causality of the association between children’s cognitive development and the cognitive stimulation that they receive. We fitted a series of genetically informative models in a large UK-population representative twin study with genotype data and measures of cognitive development and cognitive stimulation, including singing rhymes, reading books, and playing games, at the ages of 3 and 4 years.

## Methods

### Sample

The sample was drawn from the Twins Early Development Study (TEDS) that recruited 16,810 families with twin children born between 1994 and 1996 in England and Wales (Rimfeld et al. 2019). TEDS families were representative of other UK families in the 1990s; for example, 93% of the TEDS families identified as white vs. 93% of the UK’s families, 38% of TEDS mothers (47% of fathers) attained A-levels or a higher educational qualification vs. 35% of mothers in the UK population (47% of fathers), and 44% of TEDS mothers (92% of fathers) reported being employed at twins’ ages 2–4 years vs. 50% of UK mothers (91% fathers; Rimfeld et al. 2019). At the twins’ ages 3 and 4 years, 9,350 and 12,528 families were contacted respectively for data collection, of whom 6,119 and 8,198 families responded, providing data for 12,118 and 16,303 individual children at both timepoints (see the TEDS website for further details: https://www.teds.ac.uk/datadictionary/studies/returns/samples.htm). The TEDS project approval (05.Q0706/228) was granted by the ethics committee for the Institute of Psychiatry, Psychology and Neuroscience at King’s College London.

We excluded 1,797 twins because they reported severe medical problems and adverse perinatal conditions, or missed information on zygosity or gender or learning disability, or had a confirmed learning disability (see Table S1 in Supplementary Material). Sample sizes with phenotypic data ranged from a minimum of 10,634 at age 3 to a maximum of 15,314 participants at age 4 years; genotype data was available for 5,185 individuals (see Table S2 for a sample-size-by-variable breakdown and twin pair numbers).

### Measures

#### Cognitive Development

Parent-administered tests and parent-reported observations were used to assess twins’ cognitive ability at the ages 3 and 4 years. These measures have been validated against standard tests administered by trained testers (Oliver et al. 2002; Saudino et al., 1998). Specifically, nonverbal cognitive performance was assessed using age-appropriate versions of the Parent Report of Children’s Abilities (PARCA; Oliver et al. 2002; Saudino et al., 1998), while verbal ability measures were assessed by parent reports of children’s vocabulary and grammar using the CDI–III, an extension of the short form of the MacArthur Communicative Development Inventories: Words and Sentences (Fenson et al., 2007). The PARCA is an established, valid, and reliable measure of children’s early-life cognitive abilities at the ages 2, 3, and 4 years (Bayley 1993; Blaggan et al. 2014; d’Apice et al., 2019; Martin et al. 2013; McCarthy 1972; Oliver et al. 2002; Saudino et al., 1998; Price 2002; Oliver and Plomin 2007). The UK’s National Institute for Clinical Excellence (NICE 2017) uses a revised version (PARCA-R) in their developmental assessment guidelines. At age 2 years, the verbal subtest of the PARCA comprised one item on grammar (0 = talking in short, incomplete sentences to 2 = talking in long sentences and using ‘-est’ words and ‘but’). The non-verbal subtest comprised five parent-administered items, including twin block building, copying (i.e., follow the leader), drawing, paper folding, and matching. At ages 3 and 4 years, non-verbal ability was assessed with three parent-administered tests, specifically Odd One Out (16 items; e.g., child is asked to point at the two out of three images that go together), drawing (6 items; e.g., parent draws a vertical line and the child is asked to copy it), and puzzle (12 items; e.g., child is asked to identify the next image in a sequence). The parent-administered PARCA component was supplemented by parent-report items on concrete behaviours (e.g., “Does your child ever play any game with another child that involves taking turns?”; 1 = yes, 0 = no or don’t know). At each age, test scores were standardized and summed to a total score.

#### Cognitive Stimulation

At twins’ ages 3 and 4 years, parents answered the questions from the Home Observation for Measurement of the Environment (HOME) measure (Caldwell and Bradley 1984) for their first-born twin and then rated the degree to which their answer was also true for the second born twin. Answers were given on 5-point Likert scales that referred to frequency of occurrence (1 = less than once a month to 5 = almost daily) except where indicated otherwise. The HOME Inventory was designed to measure the quantity and quality of stimulation, support, and structure available in children’s home environment (Bradley 2015). Out of a total of 17 items asking parents to describe ‘twins’ early experiences’, we identified seven items that mapped onto cognitive stimulation. These seven items are listed below and capture ‘involvement in enriching activities’. They were presented to the parents under the headers “How you talk to your twins” and “Your twins’ play”. Thus, parents were asked to answer the questions from their perspective of engagement with the children.

1. Does your 1st born twin take part in nursery rhymes, simple songs, or prayers?
2. Does your 1st born twin read books or look at books with you?
3. Do you talk to your 1st born twin when you are doing household chores?
4. Does your 1st born twin have any puzzles (for example, jigsaws, puzzle boards)? Scored from none through 11 or more (5-point scale).
5. Does your 1st born twin have any children’s tapes/records/CDs (for example, of nursery rhymes, stories)? Scored from none through 11 or more (5-point scale).
6. Does your 1st born twin have any children’s books? Scored from none through 101 or more (5-point scale).
7. Does your 1st born twin play board games or card games with you (for example, “snakes and ladders”, “happy families”, “snap”)?

These items were grouped by themes into Talking and Rhymes (TR; items 1, 3, 5); Playing with Books (PB; items 2 and 6) and Playing Games (PG; items 4 and 7), which may relate differently to cognitive development with differing shares of common genetic and environmental contributions (e.g., Noble et al. 2019; Dowdall et al. 2020). Scores for these domains were derived by summing the respective items at each assessment age.

#### Polygenic Scores (PGS) for Years Spent in Education

Saliva and buccal cheek swab samples were collected when the twins were aged either 12 or 16 year. DNA was extracted and genotyped to compute polygenic scores (PGS) based on the latest genome-wide association (GWA) study for years spent in education (Lee et al. 2018; see Supplementary Methods S1 and S2 for details). The PGS were adjusted for the first ten principal components, chip, batch, and plate effects using the regression method.

### Statistical Analyses

Data preparation was carried out using STATA, and OpenMx in R (Neale et al. 2016) was used for subsequent analyses, which were preregistered at https://osf.io/5ybg4/. We departed in several ways from the preregistration. A first change was that we excluded the cognitive ability at age 2 years from our analyses, which would have violated the temporality in exploring bidirectional causal relationships.

We specified one latent factor for cognitive development (CD) with the cognitive ability test scores at ages 3 and 4 years as indicators to minimize the impacts of measurement error in preliminary genetic analyses and in the Direction of Causation biometric genetic model which can be biased by measurement error (Heath et al. 1993). Specifically, three latent factors for the cognitive stimulation domains including Talk and Rhyme (CS_TR_), Play with books (CS_PB_) and Play Games (CS_PG_) each indicated by their respective subdomain scores at ages 3 and 4 years respectively; and a latent Genetic Propensity for Education Attainment (GP_EA_) factor with the PGS for years spent in education (Lee et al. 2018) as the single indicator. To scale the latent factors, one factor loading per factor was fixed to 1. The residual variances of the factor with two indicators were constrained to be equal to identify the measurement models; the residual variance for the single-indicator factors was fixed at 0. All the observed indicator variables were residualized for age and sex. For the other genetic models (i.e., Mendelian Randomization-Direction of Causation and Cross-lagged models), we used the observed variables.

#### Phenotypic Analyses

Correlations between the latent factors of GP_EA_, CD, CS_TR_, CS_PB_ and CS_PG_ were estimated using Full Information Maximum Likelihood (FIML) estimation that utilises all the available data points for analyses (Enders and Bandalos 2001), including within-individual correlations that were constrained to be equal across birth order and zygosity, and cross-twin correlations which were estimated separately for monozygotic and dizygotic twins. Based on previous studies (Polderman et al. 2015), we expected the monozygotic cross-twin correlations to be greater than, but less than twice, the dizygotic cross-twin correlations to be consistent with models including additive genetic (A), shared/common environmental (C) and non-shared environmental (E) influences.

#### Genetic Models

The classical twin design parses the variances and covariances of variables into A, C and E components by comparing correlations between monozygotic and dizygotic twin pairs. This rests on the assumption that twin pairs who grew up together are influenced by their shared environments to the same extents, that genetic and environmental influences are distinct and that mate selection is random (Neale and Cardon 1992; Rijsdijk and Sham 2002). Univariate models were specified to estimate A, C and E influences on each of the latent phenotypes (i.e., CD, CS_TR_, CS_PB_ and CS_PG_). The variance in GP_EA_ was not similarly parsed because all its variance is assumed to reflect genetic influences.

##### Direction of Causation (DoC) Model

Three separate bivariate DoC models were specified between CD and each of the three cognitive stimulation factors CS_TR_, CS_PB_ and CS_PG_. The full DoC model specifies ACE variance components for each latent factor and two bidirectional causal paths between the two factors (i.e., CD⇆CS_TR_/CS_PB_/CS_PG_; Heath et al. 1993; Tick et al. 2016). Two further models were fitted, specifying single causal paths (i.e., CD→ CS_TR_/CS_PB_/CS_PG_ and CS_TR_/CS_PB_/CS_PG_→CD respectively). Each full, bidirectional DoC model, and the unidirectional DoC models were compared against a bivariate Cholesky decomposition, which partitions all variances and covariances into ACE components. Model comparisons were based on Chi-squared difference tests and the Akaike Information Criterion (AIC), with lower values indicating better model fit (Gillespie et al. 2003; Rasmussen et al. 2019). DoC models require ACE influences on the phenotypes to differ sufficiently (Heath et al. 1993; Rasmussen et al. 2019) but this was not the case in the current analyses. We therefore proceeded to fitting additional genetic cross-lagged models, which were not preregistered (described below).

##### Mendelian Randomization-Direction of Causation (MRDoC) Model

The MRDoC incorporates Mendelian Randomization (MR) and DoC approaches to test unidirectional causation using cross-sectional data (Minică et al. 2018). We fitted MRDoC models to test whether cognitive development causally influenced the cognitive stimulation factors at age 3 and 4 years; we could not test whether cognitive stimulation causally influenced cognitive development, because no adequate instrument variable was available. We tested the significance of the correlation between PGS based on the Lee et al. (2018) GWA study for years spent in education and cognitive development, as well as that of their respective associations within the MRDoC, to infer the suitability of PGS as an instrument variable. We encountered two difficulties with the MRDoC models. First, E influences on the cognitive development and cognitive stimulation latent factors were close to zero, which reduces the power of the MRDoC model to determine causality (Kohler et al. 2011). An alternative approach is specifying MRDoC models for observed scores of cognitive development and stimulation rather than for latent factors, with the limitation that the observed scores will include measurement error. When we used observed scores, the association between PGS and cognitive development was not significant, suggesting that PGS were not a valid instrument variable for our analyses. Thus, we concluded that the cross-lagged twin model was better suited to the current data.

##### Cross-Lagged Twin Model

We tested causal associations between observed scores of CD and CS_TR_/CS_PB_/CS_PG_ at the two timepoints of 3 and 4 years. The observed variables were used for these analyses to enable us take advantage of the longitudinal nature of the data. The genetic cross-lagged model (Burt et al. 2005) adjusts for the stability of constructs across assessment times (i.e., autoregressive paths) and for correlations between the ACE influences on each variable within each assessment age. Our extended cross-lagged twin model further controls for cross-time correlations of genetic (*r*_*A*_) and shared environmental (*r*_*C*_) factors that affect cognitive development and cognitive stimulation and might therefore confound potentially causal effects. The unique environmental cross-time correlations were restricted to zero as is commonly done when investigating causal mechanisms in twin modelling (e.g., Minică et al. 2018; Kohler et al. 2011) to facilitate statistical identification which was checked using the *mxCheckIdentification* function in OpenMx (Neale et al. 2016). The extended cross-lagged model we apply here is comparable to the approach introduced by Starr and colleagues (2023) testing cross-time causal effects between household chaos and school performance while controlling for confounding by cross-time genetic and shared environmental correlations. Our analyses applying the cross-lagged twin model and its extension were not preregistered.

## Results

Means, Standard Deviations, and Skewness of the cognitive development (CD) and cognitive stimulation variables (Talking and Rhymes, Playing with Books and Playing Games) are summarized in Table 1. The cross-twin correlations for each of the latent factors among DZ twins were lower but greater than half those in MZs suggesting A and C influences, while small E influences were indicated by the MZ cross-twin correlations being close to 1 (Table 2). The correlations between cognitive development and cognitive stimulation factors were positive and significant, ranging from moderate (*r* = 0.38, 95% CI: 0.39–0.45; for CS_PB_) to large (*r* = 0.51, 95% CI: 0.49–0.54; for CS_PG_). Similarly, the PGS, based on Lee et al.’s (2018) GWA study on years spent in full-time education, were significantly correlated with factors of cognitive development (*r* = 0.07, 95% CI: 0.03–0.10), and with cognitive stimulation (*r* = 0.09, 0.18, and 0.12 for CS_TR_, CS_PB,_ and CS_PG,_ 95% CIs: 0.05–0.22), indicating significant pleiotropy of the instrument (Table S4a-c). PGS also correlated significantly with observed scores of cognitive stimulation at ages 3 and 4 years (*r* = 0.04 to 0.13, 95% CIs: 0.01–0.17, Table S5) and with cognitive development at age 4 (*r* = 0.06, 95% CI: 0.03–0.09) but not at age 3 (*r* = 0.03, 95% CI: 0.00-0.06; Table S5).

**Table 1:** Descriptives for cognitive development and cognitive stimulation variables at age 3 and 4 years

	Cognitive development CD				Cognitive stimulation CS											
	PARCA scores				Talking-rhyme CS_TR_				Playing books CS_PB_				Playing games CS_PG_			
	N	Mean	SD	Skew	N	Mean	SD	Skew	N	Mean	SD	Skew	N	Mean	SD	Skew
Age 3	11,140	0.00	1	-0.32	10,643	0.02	1.92	-0.71	10,663	0.04	1.54	-0.21	10,634	0.02	1.56	0.20
Age 4	15,314	0.00	1	-0.23	14,015	0.03	1.94	-0.68	14,091	0.03	1.56	-0.10	14,074	0.02	1.58	-0.10

*Note* PARCA – Parent Report of Children’s Abilities; our analyses are based on standardized scores derived and provided by the TEDS team

**Table 2:** Univariate and bivariate factor correlations and univariate and standardized A, C, and E influences on latent factors of cognitive development and cognitive stimulation with 95% confidence intervals

	CD	CS_TR_	CS_PB_	CS_PG_
Univariate cross-twin correlations				
MZ twin pairs	0.99 (0.97, 1.00)	0.94 (0.92, 0.96)	0.96 (0.95, 0.98)	0.95 (0.93, 0.96)
DZ twin pairs	0.86 (0.85, 0.88)	0.79 (0.76, 0.81)	0.76 (0.74, 0.78)	0.78 (0.76, 0.80)
Bivariate correlations with CD				
Within-person	-	0.42 (0.39, 0.45)	0.38 (0.35, 0.41)	0.51 (0.49, 0.54)
Cross-twin				
MZ twin pairs	-	0.42 (0.39, 0.45)	0.37 (0.35, 0.40)	0.49 (0.47, 0.52)
DZ twin pairs	-	0.38 (0.35, 0.41)	0.34 (0.31, 0.37)	0.44 (0.41, 0.47)
Standardized univariate variance components				
h^2^	0.24 (0.20, 0.29)	0.30 (0.24, 0.37)	0.40 (0.35, 0.46)	0.33 (0.28, 0.39)
c^2^	0.74 (0.70, 0.78)	0.63 (0.58, 0.69)	0.56 (0.51, 0.61)	0.61 (0.56, 0.66)
e^2^	0.01 (0.00, 0.03)	0.06 (0.04, 0.08)	0.04 (0.02, 0.05)	0.05 (0.04, 0.07)

*Note* CD = Cognitive development latent factor, indicated by PARCA scores at ages 3 and 4 years; CS_TR_ = Cognitive Stimulation Talking and Rhyming factor, indicated by Talking and Rhyming scores at ages 3 and 4 years; CS_PB_ = Cognitive Stimulation Playing with Books factor, indicated by Playing with Books scores at ages 3 and 4 years; CS_PG_ = Cognitive Stimulation Playing Games factor, indicated by Playing Games scores at ages 3 and 4 years; MZ = monozygotic; DZ = Dizygotic; h^2^, c^2^ and e^2^ = Standardized additive genetic (A), shared (C) and non-shared (E) environmental influences

The latent factors (CD, CS_TR_, CS_PB_ and CS_PG_) were significantly influenced by latent shared environmental and additive genetic factors (56–74% and 24–40%, respectively), while non-shared environmental influences were minimal (1–6%; Table 2). The covariances between CD, and CS_TR_, CS_PB_ and CS_PG_ were predominantly attributable to shared environmental influences (ranging from 76 to 83%, Table S3), with relatively smaller contributions from latent additive genetic and non-shared environmental influences (16–20% and 1–4%, respectively). Similarly, the observed variables were mostly influenced by C components (52–63%) while non-shared influences had the smallest effects (9–20%; Table S7).

Based on the similar modes of inheritance for cognitive development and cognitive stimulation, we concluded that the DoC model was not suitable for our data to test causal effects. Likewise, the requirements for the MRDoC model were not met in our data as explained above, thus in the following we focus on the results of the cross-lagged twin models which reflect the best suited modelling approach in our case. For reasons of transparency, we report the results of the DoC models in the Supplemental Materials (see Supplementary Methods S3 for details, Tables S4a-c for model comparisons and Figure S1 for path coefficients). However, we advise against interpreting these because the similar ACE influences – which are required to be significantly different for the Direction of Causation model) – on the cognitive development and cognitive stimulation latent factors may lead to low power to determine the direction of causal effects (Health et al., 1993).

**Fig. 2:**
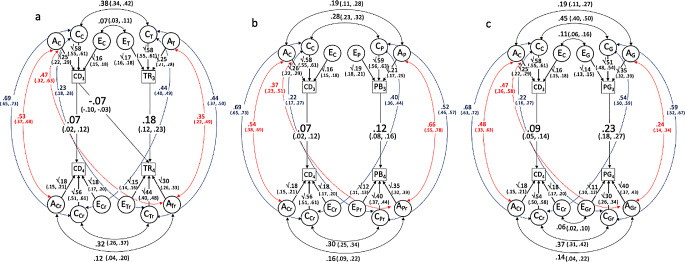
**a**-**c**: Parsimonious genetic cross-lagged models corrected for cross-time genetic and shared environmental correlations *Note* Models were fitted to observed scores, not latent factors. CD: Cognitive development; TR: Talking and rhyming; PB: Playing with books; PG: Playing games; at ages 3 and 4 years (subscripts 3 and 4, respectively). **A**, **C** and **E**: Additive genetic, shared and unique environmental influences respectively; subscripts C, T, P and G refer to CD, TR, PB and PG respectively, subscript r indicates residual effects at age 4 years. Paths which did not reach significance in the full model were restricted to zero and are not shown in the Figure to sustain clarity. Models with non-significant paths restricted to zero did not fit the data worse than the corresponding full models (Supplementary Figure S3a-c; X^2^ difference tests: X^2^_[4]_ = 6.95, *p* = 0.14; X^2^_[5]_ = 8.75, *p* = 0.12; X^2^_[3]_ = 4.94, *p* = 0.18 for models a, b and c respectively). Results from the cross-lagged models not adjusting for cross-time genetic and shared environmental correlations (following the approach by Burt et al. 2005) are presented for comparison in Figure S2a-c in the Supplemental Materials

The results from the cross-lagged twin models indicate small, yet significant direct effects of all three CS domains at age 3 on CD at 4 years (f = 0.06–0.12) as well as in the reverse direction, i.e., CD at age 3 on CS at age 4 years (g = 0.10–0.12), which suggests a bidirectional, causal relationship between cognitive development and stimulation (all model results are included in Figure S2a-c in the Supplemental Materials). However, these results are potentially confounded by cross-time genetic and shared environmental factors common to CD and CS, as these were not accounted for in the model.

We subsequently applied the extended cross-lagged twin model and found that the prospective, bidirectional associations between CD and TR, PB and PG at ages 3 and 4 years were largely due to genetic and shared environmental factors that affected both cognitive development and stimulation across ages. Figure 2a-c displays the best-fitting parsimonious model with all non-significant paths restricted to zero (the full model results are displayed in Figure S3a-c in the Supplemental Materials). The results from the extended cross-lagged model including cross-time genetic and shared environmental correlations also fit the data slightly better compared to the original model by Burt and colleagues (2005; model fit indices and model comparisons reported in Note below Figure S3). After controlling for cross-time *r*_*A*_ and *r*_*C*_, the residual direct cross-lagged effects between CD and TR, PB and PG across age 3 and 4 years were negligible (Fig. 2). An exception was a significant, negative effect of CD at age 3 on TR at age 4 (g = -0.07; 95% CI: -0.10, -0.03). That is, for children with higher cognitive development at age 3, parents reported less engagement in talking and rhyming activities one year later. This negative effect only emerged once common genetic and shared environmental factors explaining the remainder of the positive phenotypic association between children’s CD at age 3 and the TR stimulation they experience at age 4 were accounted for.

The cross-lagged effects of CD at age 3 on PB and PG at age 4 were largely due to cross-time r_A_ and r_C_ that explained 28.9% and 47.9%, respectively, of the model induced phenotypic correlation between the two constructs (for all relative contributions of the direct paths and the A, C, and E influences, see Tables S5 and S6). In the reverse direction (i.e., TR_3_/PB_3_/PG_3_→CD_4_), the cross-time r_C_ accounted for 90.5–94.2% of the overall phenotypic association, while cross-time r_A_ explained negligible variance. Regarding both directions (i.e., CD3→CS4 and CS3→CD4), the remainder of the overall phenotypic association was explained by paths through the within-time A, C, and E correlations at age 3 and the direct stability paths of either construct (0.3–17.9%).

## Discussion

Twin studies can be used to strengthen causal inferences for observed associations between two constructs by controlling for their shared genetic and environmental influences (McAdams et al. 2021). Specifically, twin studies can address whether associations between a putatively environmental exposure and developmental differences in a phenotype remain significant after accounting for the confounding effects of shared etiology (i.e., common causes; McAdams et al. 2021). Recent years have seen an explosion of novel modelling approaches that extend the classical twin design, which enable causal inferences but are yet to be systematically applied in psychological research (Erbeli et al. 2020; McAdams et al. 2021). Here, we fitted Direction-of-Causation (DoC) models (Heath, 1993), Mendelian Randomization (MR) extensions of the DoC model (Minică et al. 2018), and cross-lagged twin models (Burt et al. 2005; Starr et al. 2023) to investigate if children’s cognitive stimulation causes their cognitive development or vice versa at age 3 and 4 years.

We found bidirectional phenotypic associations between children’s cognitive development and their cognitive stimulation, which were largely explained by common genetic and shared environmental factors, rather than reflecting causal effects. We only identified one significant direct effect that might be interpreted as a causal path. Cognitive development at age 3 years had a negative effect on the level of engagement in talking and rhyming that parents reported to offer to their children at age 4 years. This – somewhat counterintuitive – finding implies that children with more advanced cognitive development at age 3 years might receive less stimulation from talking and rhyming activities one year later. However, we caution here that this small negative effect only emerged for one of the three cognitive stimulation domains and might be spurious (i.e., false positive finding due to fitting multiple models). We can speculate about two possible interpretations. First, highly intelligent children may receive less cognitive stimulation over time, perhaps because parents perceive their cognitive stimulation needs as saturated or because parents provide different, more advanced simulation but reduce rudimentary ones. Second, children who are perceived to show poor cognitive development may receive increased stimulation from their parents to help them advance and catch up. Our models suggest that this small, negative effect of cognitive development on stimulation is likely due to unique environmental factors that are thought to capture unsystematic and idiosyncratic events that make two twins in a family different from one another (Turkheimer and Waldron 2000; Plomin 2011). The exact events, experiences, or factors that drive non-shared environmental variance are yet to be determined, including environmental risk factors, for example families’ limited access to resources (i.e., low SES), substantial household chaos, and high levels of parental psychopathology (Asbury et al. 2003). Yet, our findings will need to be replicated and explored further before any conclusions about direct effects between cognitive stimulation and cognitive development can be drawn. Overall, we conclude that simultaneous, bidirectional mechanisms drive the association between cognitive development and stimulation: Genetic factors and environmental experiences shared by two children in a family explain their cognitive development as well as the cognitive stimulation that parents offer and that children evoke.

Our findings indicated that cognitively stimulating experiences are unlikely to exert causal influence on prospective cognitive development or vice versa, after controlling for the common causes of children’s cognitive development and cognitive stimulation shared by two children in a family during early childhood (i.e., genetic and shared environmental factors). Randomized controlled trials on the effectiveness of cognitive stimulation interventions to improve children’s cognitive development produced mixed results. Some studies found that play and shared book reading interventions might be suitable to improve early life cognitive development (Tachibana et al. 2012; Howard et al., 2017; Dowdall et al. 2020). For example, a meta-analysis of *n* = 54 studies on the effect of shared book reading on children’s language skills showed that effects immediately after the intervention were overall significant with modest in size ($\:\stackrel{-}{g}$ = 0.194); however, effects tended to be negligible when interventions were tested against active control groups or at follow-up assessments (Noble et al. 2019). Meta-analytic evidence also contends that effects from interventions that successfully raised children’s IQ in the short-term tend to fade out over time by about 0.10–0.13 SD per year or were not evident anymore at follow-up assessments conducted less than two years after the end of treatment (Protzko 2015; Takacs and Kassai 2019; Bailey et al. 2020).

Instead, the phenotypic association between earlier cognitive stimulation and later cognitive development is mainly driven by shared environments that likely include parenting behaviors, the family’s socioeconomic background, and characteristics of the home environment and wider neighborhood. These environmental factors are shared when they affect both children in the same way (Rijsdijk and Sham 2002). In addition, genetic factors contributed to correlations of cognitive development and stimulation within and across assessment ages. These genetic influences are likely exerted through partly genetically influenced traits, including parents’ and children’s individual characteristics, such as parental cognitive ability, educational level, and assets as well as children’s temperament, their physical health, and complex parent-child interaction processes (Tucker-Drob and Harden 2012; Brownell et al. 2016; Demange et al. 2022). However, the effects of these factors on the association between children’s cognitive development and stimulation need to be studied in depth in future research. Apart from potentially driving genetic effects on children’s cognitive development, these factors also give rise to passive and evocative gene-environment correlations (Plomin et al. 1977; Price and Jaffee 2008), which may induce bias in the variance estimates in twin models: In the presence of gene-environment correlations genetic effects are underestimated, while the shared environmental component is slightly overestimated (Verhulst and Hatemi 2013). This potential bias may in part explain the strong shared environmental effects (52-63%) and comparatively weaker genetic effects (23-40%) on children’s differences in cognitive development and cognitive stimulation in the present study. A similar pattern emerged for the etiologies of the associations between cognitive development and stimulation, which were again mainly due to shared environmental (76-84%) and additive genetic influences (16-20%). Non-shared environmental influences on cognitive development and cognitive stimulation were small and overall negligible. While this study is to our knowledge the first to estimate the heritability of cognitive stimulation and its association with cognitive development, our findings confirm that all traits are heritable, including putatively environmental measures that are genetically influenced (Krapohl et al. 2017; Plomin 1995; Polderman et al. 2015).

Our findings suggest that children’s cognitive development is not causally affected by the cognitive stimulation they experience after adjusting for genetic and environmental influences that are shared by two twins within their family. This does not imply, however, that modifying children’s cognitive stimulation or other aspects of their home learning environment cannot affect their cognitive development. Behavioural genetic studies can only describe *what is*, not *what could be*; thus, our findings do not rule out the potential of impact by interventions that target children’s cognitive stimulation.

### Limitations

Notwithstanding this study’s many strengths, including the analysis of twin and genomic data, large sample sizes, and repeated assessments of the core constructs using state-of-the-art methods, it is not without limitations. First, cognitive stimulation was assessed using seven parent-reported items, but a multi-informant approach (i.e., naturalistic home observations) with a greater number of observed variables would have improved the measures’ validity. The cognitive stimulation items were grouped into three themes (i.e., talking and rhymes, playing with books, and playing games), but alternative groupings are possible, such as differentiating cognitive stimulation activities that require initiation by the parents vs. opportunities for stimulation (i.e., toys) that twins can access without direct parent involvement. Second, data on cognitive stimulation were only collected at the twins’ ages 3 and 4 years, which made it impossible to test for meaningful changes in association with cognitive development over the longer course of childhood.

Third, our cross-lagged twin models were fitted using observed rather than latent variables. Observed variables are subject to measurement error which can obscure causal effects between cognitive stimulation and development when they are in fact present in the population. In our initial analysis strategy, we specified latent factors for the three domains of cognitive stimulation, for two of which (i.e., CS_PB_ and CS_PG_) only two observed indicators were available: for the models to be identified, their factor loadings had to be restricted. As a result, the domains of cognitive stimulation in the cross-lagged twin models included measurement error. That said, the measurement error of cognitive stimulation is likely low here because only very small proportions of nonshared environmental variance, which also capture measurement error, were evident in the univariate decompositions (e^2^ = 4–6%).

Fourth, the two core constructs might have not been sufficiently distinct from each other due to common methods biases, as cognitive stimulation and cognitive development were both reported by parents. Common methods biases can inflate the similarity between twins irrespective of their zygosity (e.g., Neale and Cardon 1992). As a result, the estimate of the shared environment may have been biased upwards for both cognitive development and cognitive stimulation, which could explain the larger shared environmental influences on cognitive development that we observed here than were reported elsewhere (e.g., Grotzinger et al. 2019; Briley and Tucker-Drob 2013), as well as the high level of shared environmental covariance between constructs. Common methods biases may have also contributed to the genetic architecture of cognitive development and cognitive stimulation not being sufficiently differentiated to meet the assumptions of DoC models (Heath, 1993; van Bergen et al. 2018).

Fifth, the association between the PGS for years spent in education (Lee et al. 2018) and cognitive development was small, which indicates that the PGS was a weak instrument for our phenotypic measure of cognitive development that can lead to an overestimation of causal effects (Burgess and Thompson 2011). The weak associations between the PGS and the phenotype might in this case be due to developmental effects, since the PGS was aimed to capture educational achievement in adulthood. Cognitive development in early childhood differs as a construct from adult educational achievement, is less strongly influenced by genetic factors, and exhibits considerable instability over time compared to cognitive ability in adulthood (von Stumm et al. 2023; Breit et al. 2024; Petrill et al. 2004). As a result, the MRDoC analysis strategy we had preregistered was not viable, and we fitted cross-lagged twin models instead that require longitudinal data.

In sum, maximising the explanatory power of complex genetically informed statistical models, like the ones we attempted to fit here, is often difficult. Leveraging the theoretical value of DoC and MRDoC models is only possible if empirical data are available that meet the essential model assumptions, including sufficiently different aetiologies and valid instrument variables. Finding better synergies between statistical models and available data than reflected in the current study should be a priority for future research.

## Conclusions

Across different genetically sensitive study designs, we found that genetic and shared environmental factors drive the association between children’s differences in cognitive development and in the cognitive stimulation that they receive from their parents during the early years. While our study did not produce evidence for causal effects between children’s cognitive development and stimulation, such effects may exist for other traits or emerge at later time points during children’s development.

## Electronic Supplementary Material

Below is the link to the electronic supplementary material.

Supplementary Material 1

## Data Availability

Open Practices Statement: The data for this study can be accessed here: https://www.teds.ac.uk/researchers/teds-data-access-policy. The study materials can be accessed here: https://teds.ac.uk/datadictionary/home.htm The analysis code for this study is available on github: https://github.com/Kaoginni/MRDoC-and-Cross-lagged-model-adjusting-for-cross-time-rA-and-rC/blob/main/Oginni%2C%20Starr%20and%20von%20Stumm%202024.R. There is a preregistration for this study: available on the OSF: https://doi.org/10.17605/OSF.IO/D7Y59.
