# Supplementary material for: Do Children Cause the Cognitive Stimulation they Receive? Modelling the Direction of Causality
Source: Behav Genet. 2024 Sep 10;54(6):443–55. doi: 10.1007/s10519-024-10195-w (PMC11564400; doi:10.1007/s10519-024-10195-w)
Supplement: Supplementary file 1 — Supplementary Material 1 [file 10519_2024_10195_MOESM1_ESM.docx]

**Supplementary Materials**

**Do children cause the cognitive stimulation they receive?**

**Modelling the direction of causality**

**Table of Contents**

Methods

S1. Genotyping and quality control

S2. Creating polygenic scores using LDpred

S3. Direction of Causation (DoC) models

Results

Table S1: Participants excluded from analyses at ages 3 and 4 years

Table S2: Number of participants (and complete twin pairs) with complete data per variable

Table S3: Standardized ACE decomposition of the covariances between cognitive development and the cognitive stimulation factors

Tables S4a-c: Exploratory model comparisons for bidirectional relationships between Cognitive development and Cognitive stimulation domains (CS_TR_, CS_PB_, CS_PG_)

Table S5: Phenotypic correlations between observed variables at ages 3 and 4 years

Table S6: Phenotypic correlations across time between cognitive development and cognitive stimulation domains and relative contributions of causal paths and A, C and E influences from the cross lagged models

Table S7: Standardized ACE influences on the variances and covariances of the observed scores across age 3 and 4 years

Figure S1: Mendelian Randomisation-Direction of Causation models testing for causal effects of Cognitive Development on the Cognitive Stimulation domains at ages 3 and 4 years

**Methods S1. Genotyping and quality control**

DNA from 8,122 individuals was extracted from saliva and buccal cheek swab samples and hybridized to HumanOmniExpressExome-8v1.2 genotyping arrays at the Institute of Psychiatry, Psychology and Neuroscience Genomics & Biomarker Core Facility. Raw image data were pre-processed in GenomeStudio according to Illumina Exome Chip SOP v1.4. (<http://confluence.brc.iop.kcl.ac.uk:8090/display/PUB/Production+Version%3A+Illumina+Exome+Chip+SOP+v1.4>). Prior to genotype calling, 919 multimapping SNPs and 501 samples with call rate <0.95 were removed. Following initial QC, the program ZCALL was used to augment genotype calling.

DNA from 3,747 individuals was extracted from buccal cheek swabs and genotyped at Affymetrix, Santa Clara, California, USA. From the extracted DNA samples, 3,665 samples were successfully hybridized to AffymetrixGeneChip 6.0 SNP genotyping arrays ([http://www.affymetrix.com/support/technical/datasheets/genomewide_snp6_datashe et.pdf](http://www.affymetrix.com/support/technical/datasheets/genomewide_snp6_datashe%20et.pdf)) using experimental protocols recommended by the manufacturer.

Raw image data were pre-processed at the Wellcome Trust Sanger Institute, Hinxton, UK for genotyping as part of the Wellcome Trust Case Control Consortium 2 (<https://www.wtccc.org.uk/ccc2/>). All pre-processing was conducted according to the manufacturer’s guidelines (http://www.affymetrix.com/support/downloads/manuals/genomewidesnp6_manual.pdf). After initial QC, the program CHIAMO was used for genotype calling (https://mathgen.stats.ox.ac.uk/genetics_software/chiamo/chiamo.html).

After initial quality control, the same quality control was performed on samples from each of the platforms (Illumina and Affymetrix) separately using PLINK (Purcell et al., 2007), R(Team, 2015) and BCFtools(Li, 2011) and EIGENSOFT(Patterson, Price, & Reich, 2006; Price et al., 2006).

DNA samples were excluded from subsequent analyses on the basis of call rate (<0.98), suspected non-European ancestry, the presence of severe medical or psychiatry problems or severe medical complications during early gestation and relatedness other than dizygotic twin status. SNPs were excluded if the minor allele frequency was <0.5%, if more than 2% of genotype data were missing, or if the Hardy Weinberg *p*-value was lower than 10^-5^. Non-autosomal markers and indels were also removed. Associations between SNPs and the platform, batch, plate or well on which samples were genotyped were calculated; SNPs with an effect p-value < 10-4 were excluded.

A total sample of 10,346 samples, including 7,026 unrelated individuals; 3,320 additional individuals had a genotyped dizygotic co-twin. Genotype data following quality control were available for 4,776 individuals and 559,772 SNPs from the illumine array and 2,250 individuals and 635,269 SNPs from the Affymetrix array.

Genomewide genotypes from the two arrays were separately phased using EAGLE2 (Loh et al., 2016) and imputed using the Haplotype Reference Consortium (McCarthy et al., 2016) using the Positional Burrows-Wheeler Transform method (Durbin, 2014) and the imputation software Minimac3 1.0.13 (Fuchsberger, Abecasis, & Hinds, 2015), which are available from the Michigan Imputation Server (https://imputationserver .sph.umich.edu). A series of quality checks were performed before merging data from the two arrays, and variants with info <0.75 were excluded and SNPs that were non-overlapping between platforms were removed.

After merging, minor allele frequency differences were tested for between platforms and SNPS with an effect p-value <10^-4^ were removed. Those SNPs with a Hardy-Weinberg p-value >10^-5^ were also removed. Following these criteria, 7,363,646 genotyped and well-imputed SNPs were retained for analyses. Only unrelated individuals were included in the present analyses. To ease high computational demands of the software LDpred (Vilhjálmsson et al., 2015) for polygenic scoring in large samples, we further excluded SNPs with info <1, leaving 515,100 SNPs for analysis.

**Methods S2. Creating polygenic scores using LDpred**

Polygenic scores were calculated using LDpred, which has been shown to outperform predictive accuracy of the conventional clumping and p-value thresholding approach (Vilhjálmsson et al., 2015). Here, a posterior effect size is derived for each SNP by re-weighting the original summary statistic coefficient by the relative influence of a SNP given its level of linkage disequilibrium with surrounding SNPS and a prior on the effect size of each SNP. The prior is based on the heritability of the trait and the fraction of markers assumed to casually influence the trait. GPS is then calculated as the sum of the trait-increasing alleles weighted by their posterior effect size estimate. Unlike the conventional clumping and thresholding approach, LDpred retains all SNPs common between GWA summary statistics and genotype data in the target sample.

For the present study we applied a causal fraction of 1, which assumes that all SNPs contribute to the development of the trait. Due to the high computational demand of LDpred, especially in larger sample sizes including many SNPs, we made further restrictions to our analyses, only including the 515,100 SNPs that were perfectly imputed (info score of 1) to reduce analytical load. Only genotypes of unrelated individuals were used to estimate LD structure in our sample because levels of LD are considerably higher in relatives compared to unrelated individuals (Vattikuti, Guo, & Chow, 2012).

**Method S3. Direction of Causation (DoC) models**

As the estimates of the A, C, and E influences derived from the univariate Cholesky models for the latent factors of interest (cognitive development – CD and all three domains of cognitive stimulation – CS_TR_, CS_PB_ and CS_PG_ i.e., Talk-Rhyming, Playing with Books and Playing Games respectively) were not clearly distinct or non-identical (Heath et al., 1993; Rasmussen et al., 2019), we only specified the twin Direction of Causation (DoC) models as part of exploratory analyses. For these, each DoC model comprised two sets of ACE influences unique to each latent factor and two reciprocal causal paths between both latent factors (e.g., CA← →CS_TR_; Heath et al., 1993; Tick et al., 2016). Two submodels, each with a unidirectional causal path, were then specified (e.g., CA→CS_TR_ and CA←CS_TR_). Each full (bidirectional) DoC model and its (unidirectional) submodels were compared against a corresponding bivariate Cholesky model using Chi-squared tests and the Akaike Information Criterion (AIC) for nested (models with unidirectional causal paths) and non-nested (model with bidirectional causal paths) models, respectively, with lower fit indices indicating better fit (Gillespie et al., 2003; Rasmussen et al., 2019). The results of these analyses are reported below in Tables S4a-c.

**RESULTS. Table S1: Participants excluded from analyses at ages 3 and 4 years**

|  | Age 3 years  n=12118 | | Age 4 years  n=16303 | |
| --- | --- | --- | --- | --- |
|  | n | % | n | % |
| General exclusion | 358 |  | 524 |  |
| Medical exclusion | 113 |  | 173 |  |
| Perinatal outliers | 202 |  | 304 |  |
| Unknown Zygosity | 8 |  | 30 |  |
| Learning disability | 632 |  | 470 |  |
| Total excluded^a^ | 903 |  | 894 |  |
| Total eligible | 11215 |  | 15409 |  |

^a^The sums of excluded participants are greater than the total excluded due to overlap between the exclusion categories.

**RESULTS. Table S2: Number of participants (and complete twin pairs) with data available per variable**

|  | Total sample | Complete MZ  twin pairs | Complete DZ  twin pairs |
| --- | --- | --- | --- |
|  | n | n | n |
| Age 3 years |  |  |  |
| PARCA score | 11140 | 1855 | 3502 |
| Talk-Rhyme^a^ | 10643 | 1745 | 3297 |
| Playing with Books^b^ | 10663 | 1751 | 3307 |
| Playing Games^c^ | 10634 | 1740 | 3299 |
|  |  |  |  |
| Age 4 years |  |  |  |
| PARCA scores | 15314 | 2590 | 4903 |
| Talk-Rhyme^a^ | 14015 | 2354 | 4458 |
| Playing with Books^b^ | 14091 | 2367 | 4488 |
| Playing Games^c^ | 14074 | 2361 | 4484 |
|  |  |  |  |
| PGS | 5185 | -^d^ | 1666 |

*Note:* PARCA - Parent Report of Children’s Abilities; MZ – monozygotic; DZ – dizygotic; PGS – Polygenic scores for years spent in education.

^a^Sum of three cognitive stimulation items.

^b^Sum of two cognitive stimulation items.

^c^Sum of two cognitive stimulation items.

^d^Only one of a monozygotic twin pair was genotyped as each pair is 100% genetically similar.

**RESULTS. Table S3: Standardized ACE decomposition of the covariances between cognitive development and the cognitive stimulation factors**

|  | CD-CS_TR_ | CD-CS_PB_ | CD-CS_PG_ |
| --- | --- | --- | --- |
| Standardized component |  |  |  |
| h^2^ | 0.16  (0.09, 0.23) | 0.18  (0.11, 0.26) | 0.20  (0.15, 0.26) |
| c^2^ | 0.84  (0.78, 0.90) | 0.80  (0.74, 0.86) | 0.76  (0.71, 0.81) |
| e^2^ | 0.01  (-0.01, 0.03) | 0.02  (0.00, 0.04) | 0.04  (0.02, 0.05) |

*Note:* ACE = Additive genetic, and shared and non-shared environmental influences respectively; h^2^ c^2^ and e^2^ = Standardized A, C and E influences respectively, CD=Cognitive Development factor, CS_TR_, CS_PB_ and CS_PG_=Cognitive Stimulation (Talk-Rhyming, Playing with Books and Playing Games domains respectively)

**RESULTS.**

**Table S4a: Exploratory model comparisons for bidirectional relationships between cognitive development and cognitive stimulation (Talking and rhyming domain - CS_TR_)**

| Model | ep | -2LL | df | AIC | Δ−2LL | Δdf | *p* |
| --- | --- | --- | --- | --- | --- | --- | --- |
| Cholesky | 25 | 115690.0 | 51087 | 115740.0 |  |  |  |
| No relation | 22 | 116429.9 | 51090 | 116473.0 | 739.05 | 3 | <0.001 |
| CD → CS_TR_ | 23 | 115700.5 | 51089 | 115746.5 | 10.53 | 2 | 0.005 |
| CS_TR_ → CD | 23 | 115755.8 | 51089 | 115801.8 | 65.84 | 2 | <0.001 |
| **CD⇆CS_PG_** | **24** | **115691.7** | **51088** | **115739.7** |  |  |  |

*Note:* The models with the best fit are highlighted in bold.

**Table S4b:** **Exploratory model comparisons for bidirectional relationships between cognitive development and cognitive stimulation (Playing with books domain - CS_PB_)**

| Model | ep | -2LL | df | AIC | Δ−2LL | Δdf | *p* |
| --- | --- | --- | --- | --- | --- | --- | --- |
| Cholesky | 25 | 119966.5 | 51183 | 120016.5 |  |  |  |
| No relation | 22 | 120633.4 | 51186 | 120677.4 | 666.83 | 3 | <0.001 |
| **CD → CS_PB_** | **23** | **119969.5** | **51185** | **120015.5** | **2.93** | **2** | **0.23** |
| CS_PB_ → CD | 23 | 120016.9 | 51185 | 120062.9 | 50.36 | 2 | <0.001 |
| CD⇆CS_PG_ | 24 | 119967.3 | 51184 | 120015.3 | 0.79 | 1 |  |

*Note:* The models with the best fit are highlighted in bold.

**Table S4c:** **Exploratory model comparisons for bidirectional relationships between cognitive development and cognitive stimulation (Playing games domain - CS_PG_)**

| Model | ep | -2LL | df | AIC | Δ−2LL | Δdf | *p* |
| --- | --- | --- | --- | --- | --- | --- | --- |
| Cholesky | 25 | 118755.5 | 51137 | 118805.5 |  |  |  |
| No relation | 22 | 120018.8 | 51140 | 120062.8 | 1263.32 | 3 | <0.001 |
| **CD → CS_PG_** | **23** | **118760.6** | **51139** | 118806.6 | **5.15** | **2** | **0.08** |
| CS_PG_ → CD | 23 | 118788.6 | 51139 | 118834.6 | 33.18 | 2 | <0.001 |
| CD⇆CS_PG_ | 24 | 118759.6 | 51138 | 118807.6 | 4.15 | 1 |  |

*Note:* The models with the best fit are highlighted in bold.

**RESULTS. Table S5: Phenotypic correlations between observed variables at ages 3 and 4 years**

| Variables | PRSEA  (1) | PARCA_3_  (2) | TR_3_  (3) | PB_3_  (4) | PG_3_  (5) | PARCA_4_  (6) | TR_4_  (7) | PB_4_  (8) | PG_4_  (9) |
| --- | --- | --- | --- | --- | --- | --- | --- | --- | --- |
| 1. | 1 |  |  |  |  |  |  |  |  |
| 2. | 0.03  (0.00, 0.06) | 1 |  |  |  |  |  |  |  |
| 3. | 0.07  (0.04, 0.10) | 0.24  (0.22, 0.26) | 1 |  |  |  |  |  |  |
| 4. | 0.13  (0.10, 0.16) | 0.21  (0.19, 0.23) | 0.41  (0.39, 0.43) | 1 |  |  |  |  |  |
| 5. | 0.09  (0.06, 0.12) | 0.32  (0.30, 0.34) | 0.30  (0.27, 0.32) | 0.38  (0.36, 0.40) | 1 |  |  |  |  |
| 6. | 0.06  (0.03, 0.09) | 0.58  (0.56, 0.59) | 0.25  (0.23, 0.28) | 0.23  (0.21, 0.25) | 0.27  (0.25, 0.29) | 1 |  |  |  |
| 7. | 0.04  (0.01, 0.07) | 0.20  (0.18, 0.22) | 0.48  (0.46, 0.50) | 0.31  (0.29, 0.33) | 0.27  (0.24, 0.29) | 0.21  (0.19, 0.23) | 1 |  |  |
| 8. | 0.13  (0.10, 0.17) | 0.21  (0.19, 0.23) | 0.33  (0.31, 0.35) | 0.56  (0.54, 0.58) | 0.34  (0.32, 0.36) | 0.22  (0.20, 0.24) | 0.38  (0.36, 0.39) | 1 |  |
| 9. | 0.08  (0.05, 0.11) | 0.29  (0.27, 0.31) | 0.31  (0.29, 0.34) | 0.39  (0.37, 0.41) | 0.55  (0.53, 0.57) | 0.28  (0.26, 0.29) | 0.35  (0.33, 0.37) | 0.40  (0.38, 0.42) | 1 |

*Note:* PRSEA = polygenic score for educational attainment; PARCA= Parent Report of Children's Abilities; TR=Talking and Rhymes; PB=Playing with Books; PG=Playing Games; subscripts 3, 4 indicate scores at ages 3 years and 4 years respectively; Coefficients in green font indicate cross-time correlations between PARCA, TR, PB and PG at ages 3 and 4 years.**RESULTS.** **Table S6: Phenotypic correlations across time between cognitive development and cognitive stimulation domains and relative contributions of causal paths and A, C and E influences from the parsimonious cross lagged models adjusted for cross-time A and C correlations**

|  | Observed correlation (rPh) | Causal path coefficient | Cross-time rA | Cross-time rC | Path through within-time A/C/E correlation at age 3 years and stability path^a^ | | | % of rPh due to causal path | % of rPh due to cross-time rA | % of rPh due to cross-time rC | % of rPh due to within-time A/C/E correlations at age 3 years and stability path | | |
| --- | --- | --- | --- | --- | --- | --- | --- | --- | --- | --- | --- | --- | --- |
|  |  |  |  |  | A | C | E |  |  |  | A | C | E |
| CD_3_→TR_4_ | 0.20  (0.18, 0.22) | -0.067 | 0.129 | 0.115 | - | 0.040 | 0.002 | - ^b^ | - ^b^ | - ^b^ | - ^b^ | - ^b^ | - ^b^ |
| CD_3_→PB_4_ | 0.21  (0.19, 0.23) | - | 0.110 | 0.108 | 0.005 | 0.020 | - | - | 45.14 | 44.41 | 2.26 | 8.18 | - |
| CD_3_→PG_4_ | 0.29  (0.27, 0.31) | - | 0.149 | 0.090 | 0.013 | 0.056 | 0.004 | - | 47.93 | 28.92 | 4.06 | 17.89 | 1.19 |
| TR_3_→CD_4_ | 0.25  (0.23, 0.28) | - | - | 0.253 | - | 0.016 | 0.001 | - | - | 93.86 | - | 5.82 | 0.31 |
| PB_3_→CD_4_ | 0.23  (0.21, 0.25) | - | - | 0.231 | 0.003 | 0.011 | - | - | - | 94.16 | 1.26 | 4.57 | - |
| PG_3_→CD_4_ | 0.27  (0.25, 0.29) | - | - | 0.286 | 0.005 | 0.023 | 0.002 | - | - | 90.47 | 1.67 | 7.37 | 0.49 |

*Note:* CD=Cognitive Development; TR=Talking and Rhymes; PB=Playing with Books; PG=Playing Games; subscripts 3, 4 indicate scores at ages 3 years and 4 years respectively; A and C contributions to cross-time correlations are determined by multiplying standardised path coefficients following path tracing rules and tracing from CD_3_ and TR_3_/PB_3_/PG_3_ through the additive and shared environmental (A_C_ and C_C_ respectively) components paths at age 3 years, through the cross-lag cross-time correlation paths and the additive genetic and shared environmental (A_Cr_ and C_Cr_) components paths (at age 4 years) to TR_4_/PB_4_/PG_4_ and CD_4_ paths. Note that cross-time non-shared environmental (E) correlations are fixed to 0 for identification; sums of the correlation components are sometimes greater than the phenotypic correlations due to rounding up errors.

^a^CD_3_→CD_4_ stability path when TR_3_/PB_3_/PG_3_ is the predictor and TR_3_→TR_4_/PB_3_→PB_4_/PG_3_→PG_4_ stability paths when CD_3_ is the predictor; ^b^Percentages not computed because the negative coefficients distort the proportions.

**RESULTS. Table S7: Standardized ACE influences on the variances and covariances of the observed scores across age 3 and 4 years**

|  | PARCA_3_ (1) | PARCA_4_ (2) | TR_34_ (3) | PB_34_ (4) | PG_34_ (5) |
| --- | --- | --- | --- | --- | --- |
| h^2^ |  |  |  |  |  |
| 1. | 0.23  (0.19, 0.27) |  |  |  |  |
| 2. | 0.23  (0.18, 0.29) | 0.17  (0.13, 0.21) |  |  |  |
| 3. | 0.11  (-0.01, 0.22) | 0.19  (0.08, 0.30) | 0.31  (0.27, 0.34) |  |  |
| 4. | 0.17  (0.05, 0.30) | 0.15  (0.04, 0.27) | 0.11  (0.05, 0.17) | 0.28  (0.24, 0.32) |  |
| 5. | 0.22  (0.14, 0.30) | 0.17  (0.08, 0.26) | 0.04  (-0.03, 0.10) | 0.15  (0.10, 0.21) | 0.40  (0.36, 0.43) |
|  |  |  |  |  |  |
| c^2^ |  |  |  |  |  |
| 1. | 0.60  (0.56, 0.63) |  |  |  |  |
| 2. | 0.75  (0.71, 0.79) | 0.63  (0.60, 0.67) |  |  |  |
| 3. | 0.88  (0.78, 0.97) | 0.82  (0.73, 0.90) | 0.57  (0.54, 0.60) |  |  |
| 4. | 0.79  (0.68, 0.90) | 0.83  (0.73, 0.93) | 0.81  (0.75, 0.86) | 0.58  (0.55, 0.61) |  |
| 5. | 0.75  (0.67, 0.82) | 0.82  (0.73, 0.90) | 0.90  (0.85, 0.96) | 0.78  (0.73, 0.83) | 0.52  (0.48, 0.55) |
|  |  |  |  |  |  |
| e^2^ |  |  |  |  |  |
| 1. | 0.17  (0.16, 0.19) |  |  |  |  |
| 2. | 0.01  (0.00, 0.03) | 0.20  (0.18, 0.21) |  |  |  |
| 3. | 0.02  (-0.02, 0.05) | 0.00  (-0.04, 0.03) | 0.12  (0.11, 0.13) |  |  |
| 4. | 0.04  (0.00, 0.07) | 0.02  (-0.02, 0.05) | 0.09  (0.07, 0.10) | 0.14  (0.13, 0.15) |  |
| 5. | 0.03  (0.01, 0.05) | 0.01  (-0.01, 0.04) | 0.06  (0.04, 0.08) | 0.06  (0.05, 0.08) | 0.09  (0.08, 0.10) |

*Note:* PARCA= Parent Report of Children's Abilities; TR=Talking and Rhymes; PB=Playing with Books; PG=Playing Games; subscripts 3, 4 and 34 indicate scores at ages 3 years, 4 years and the sum of scores at both ages; h^2^, c^2^ and e^2^=standardised additive genetic and shared and unique environmental influences respectively.


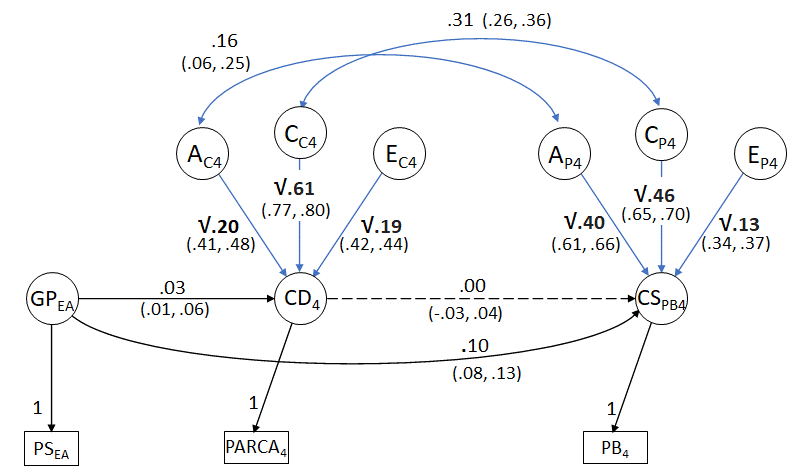


bii.


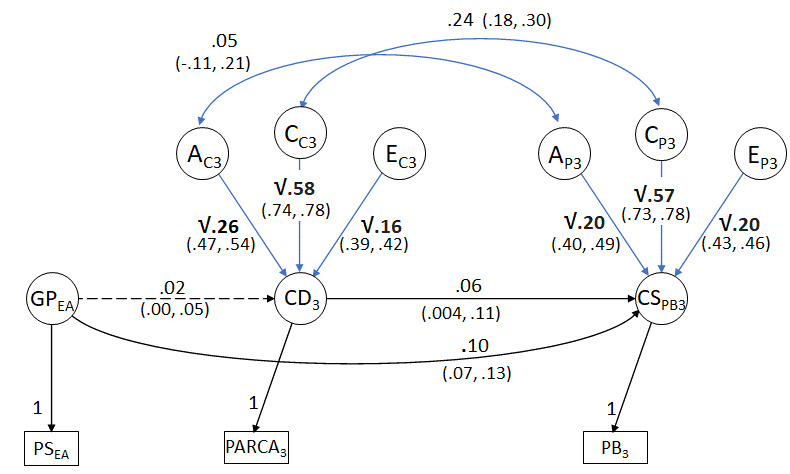


bi.


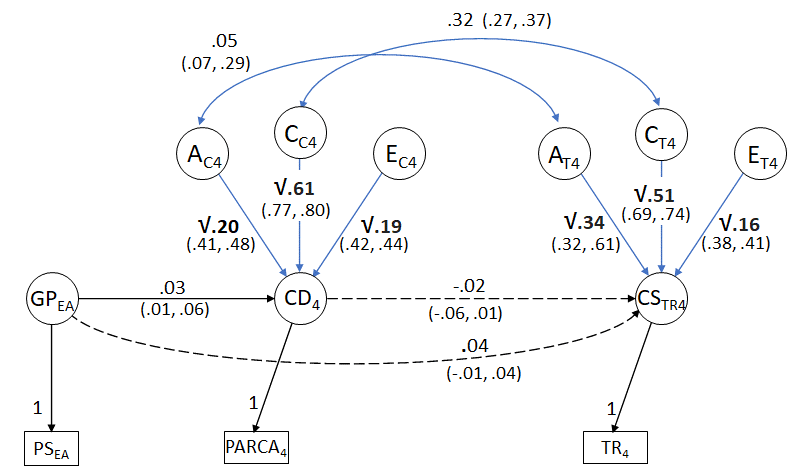


aii.


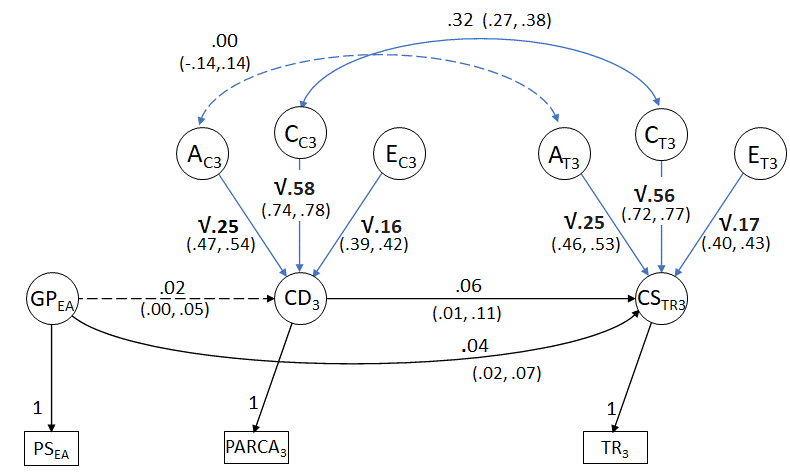


ai.


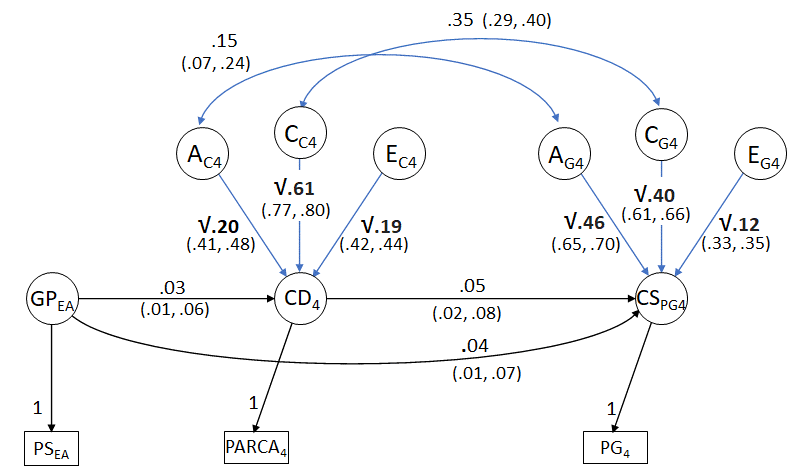


cii.


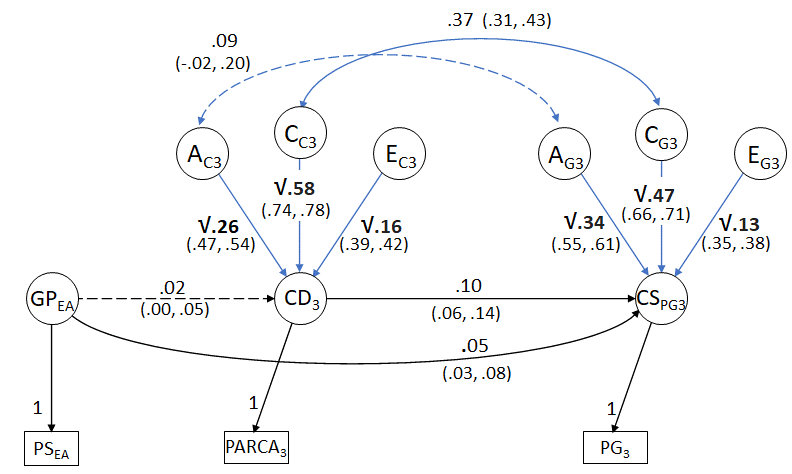


ci.

**Figure S1: Mendelian Randomisation-Direction of Causation models testing for causal effects of Cognitive Development on the Cognitive Stimulation domains at ages 3 and 4 years**

*Note:* Mendelian Randomisation-Direction of Causation models testing for causal effects of CD (Cognitive Development) on the domains of Cognitive Stimulation (CS_TR_, CS_PB_ and CS_PG_ - Talking and rhyming [TR], Playing with books [PB] and Playing games [PG]). PS_EA_, GP_EA_=Polygenic Score and Genetic Propensity for Educational Attainment, A, C and E – additive genetic, and shared and non-shared environmental influences on Cognitive Development (subscript C) and domains of Cognitive Stimulation (subscripts T, P and G); all relationships are depicted at ages 3 and 4 years (subscripts 3 and 4 respectively).


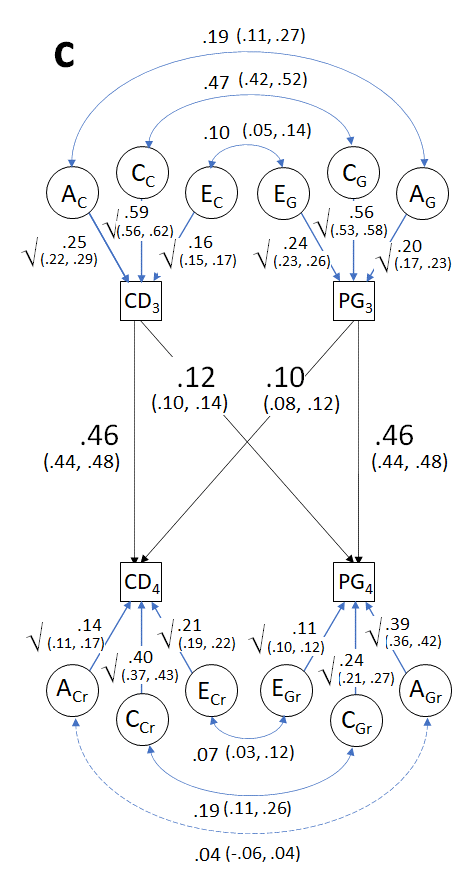

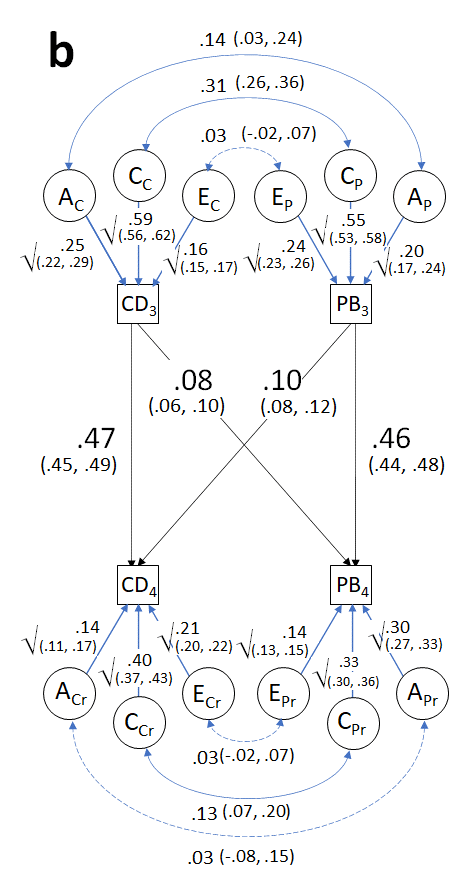

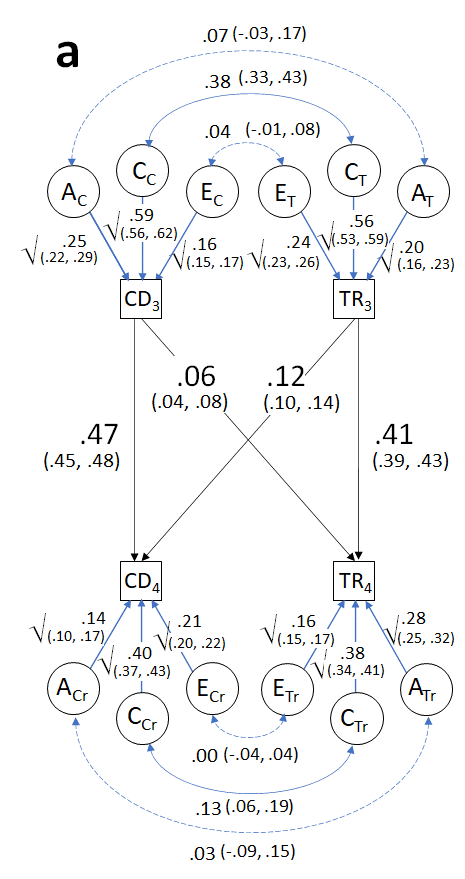


**Figure S2a-c: Genetic cross-lagged models for cognitive development and domains of cognitive stimulation**

*Note:* Models were fitted to observed scores, not latent factors. CD = Cognitive Development; TR = Talking and rhyming; PB = Playing with books; PG = Playing games; subscripts 3 and 4 = at ages 3 and 4 years, respectively; A, C, and E = Additive genetic, shared, and unique environmental influences; subscripts C, T, P and G refer to CD, TR, PB and PG respectively; subscript r indicates residual effects at age 4 years


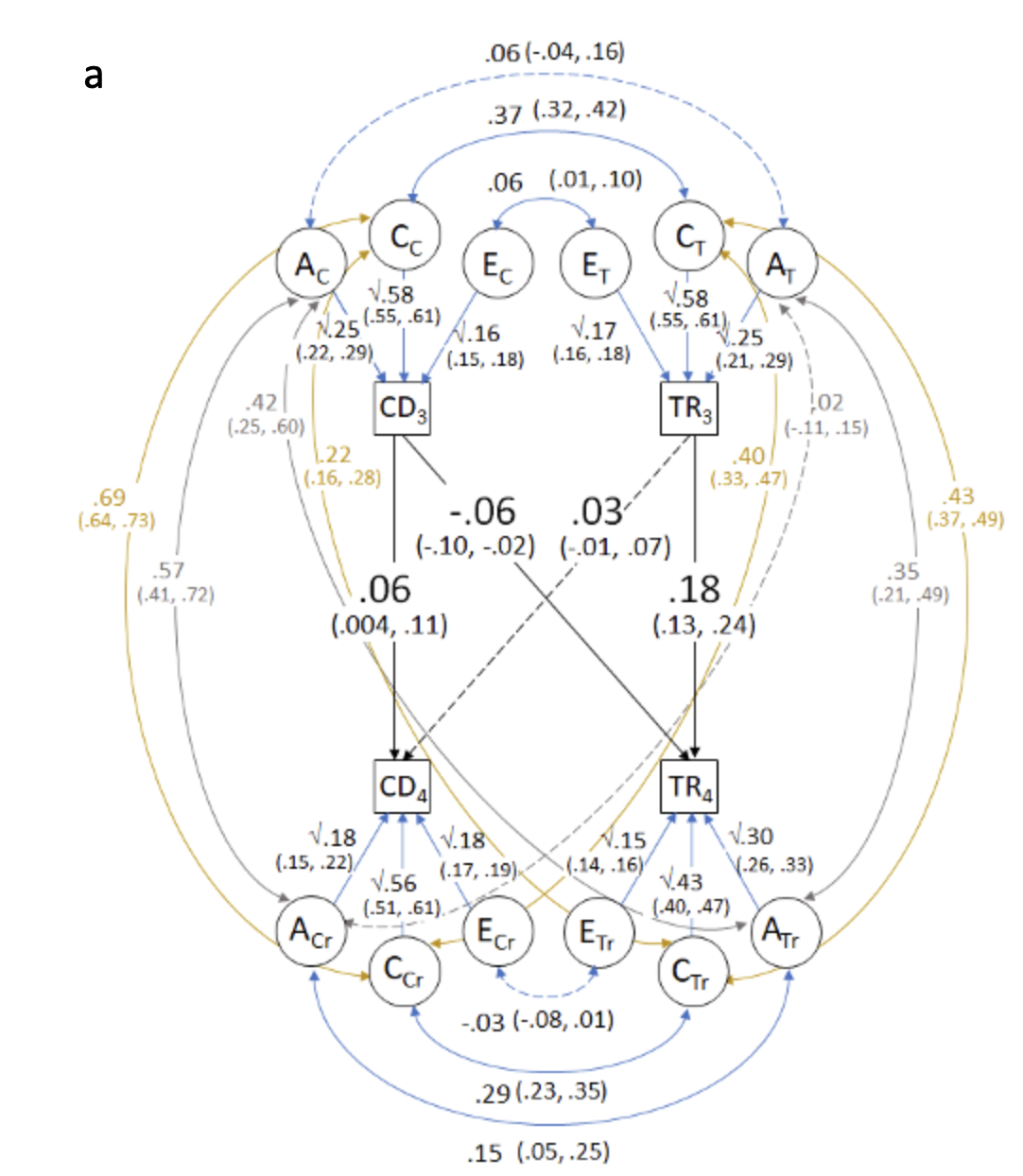

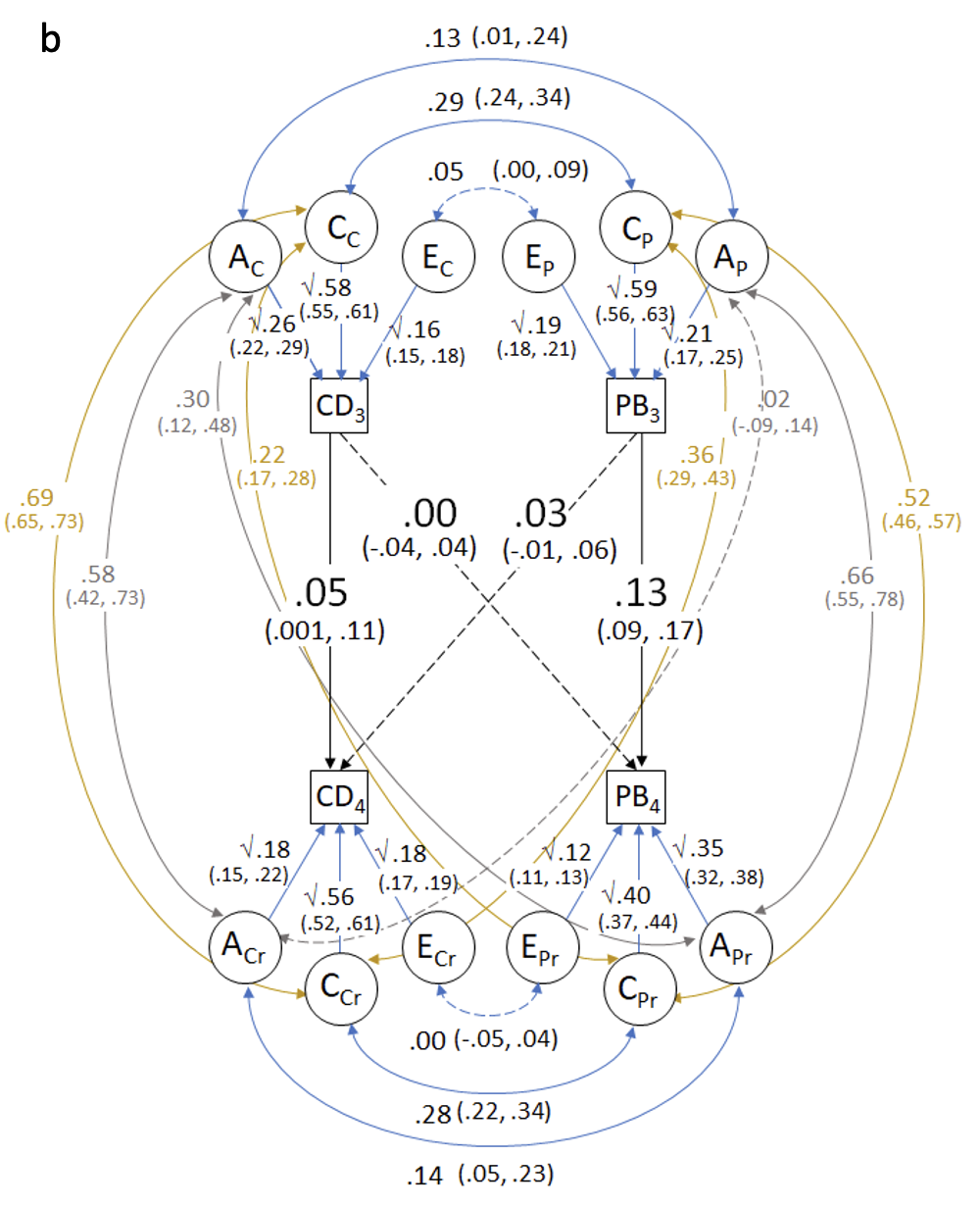


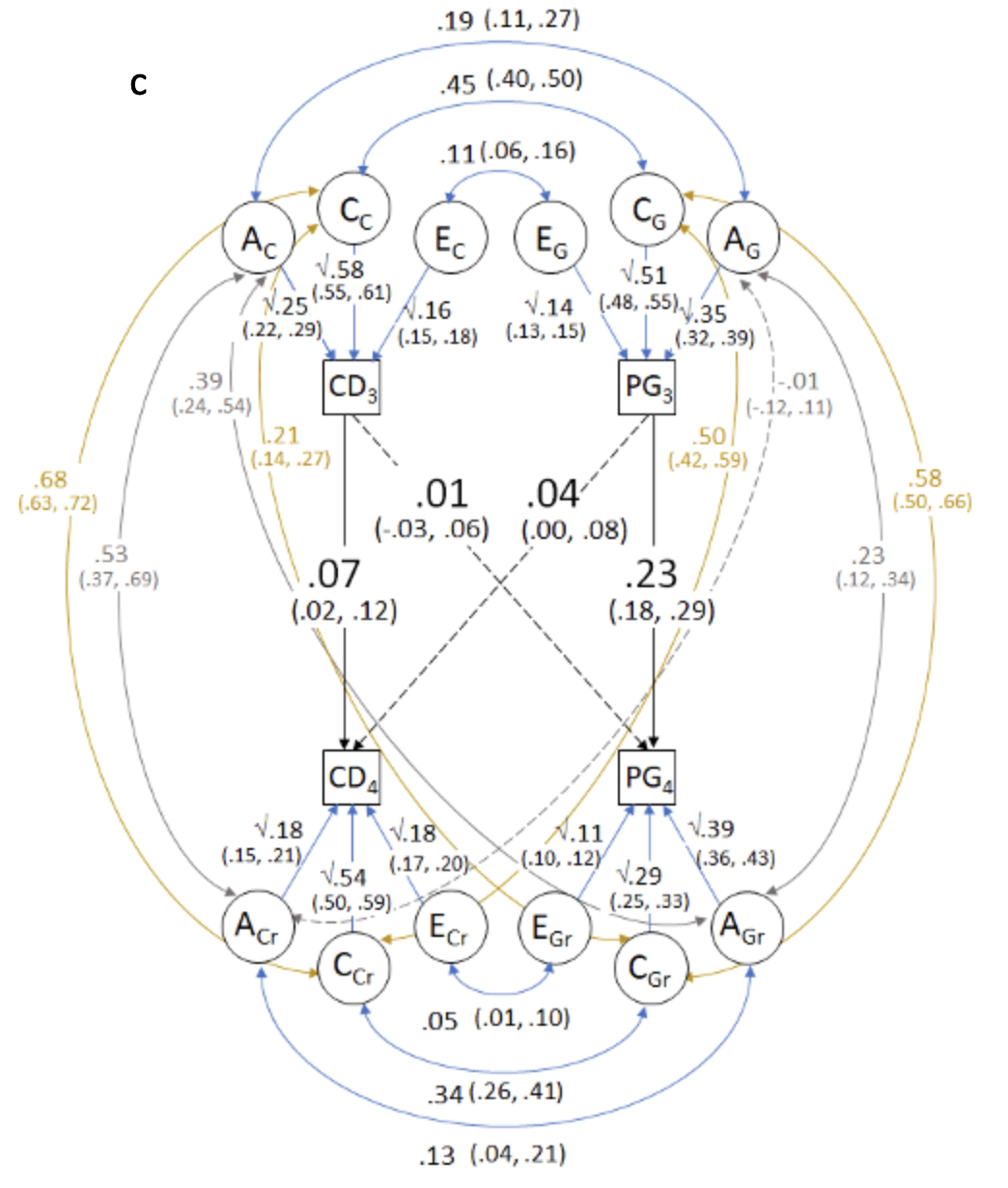


**Figure S3a-c: Genetic cross-lagged models for cognitive development (CD) and domains of cognitive stimulation**

*Note.* TR: Talking and rhyming; PB: Playing with books; PG: Playing games; at ages 3 and 4 years [subscripts 3 and 4 respectively]; a-c respectively). A, C and E: Additive genetic, shared and unique environmental influences respectively; subscripts C, T, P and G refer to CD, TR, PB and PG respectively, subscript r indicates residual effects at age 4 years. In this set of models, the stability paths (CD_3_→CD_4_ and TR_3_/PB_3_/PG_3_→TR_4_/PB_4_/PG_4_) and the cross-lagged paths (CD3→TR_4_/PB_4_/PG_4_ and TR_3_/PB_3_/PG_3_→CD_4_) are adjusted for cross-time correlations between A and C influences at ages 3 and 4 years (grey and brown lines respectively). These models have significantly better fit compared to the models without these corrections displayed in Figure S2a-c (ꭓ^2^_[8]_=733.78; 988.50 and 739.88 for TR, PB and PG respectively; *p*<0.001 for all model comparisons).
